# Profiles of bacterial communities and environmental factors associated with proliferation of malaria vector mosquitoes within the Kenyan Coast

**DOI:** 10.1099/acmi.0.000606.v4

**Published:** 2023-08-17

**Authors:** Josphat Mutinda, Samuel Mwakisha Mwamburi, Kennedy Omondi Oduor, Maurice Vincent Omolo, Regina Mongina Ntabo, James Muhunyu Gathiru, Joseph Mwangangi, James O. M. Nonoh

**Affiliations:** ^1^​ Kenyatta University, P.O. Box 43844-00100, Nairobi, Kenya; ^2^​ Kenya Marine and Fisheries Research Institute, P.O Box 81651- 80100, English Point, Mkomani, Mombasa, Kenya; ^3^​ Masinde Muliro University of Science and Technology, Centre for African Medicinal and Nutritional Flora and Fauna (CAMNFF), P.O Box 190-50100, Kakamega, Kenya; ^4^​ Kenya Medical Research Institute (KEMRI), Centre for Geographic Medicine Research - Coast, Kilifi P.O. Box 428, Kilifi – 80108, Kenya; ^5^​ Maseno University, Private Bag, Maseno, Kenya

**Keywords:** bacterial communities, physico-chemical factors, breeding sites, oviposition sites, *Anopheles gambiae*

## Abstract

**Background.:**

Since *Anopheles* mosquitoes which transmit and maintain the malaria parasite breed in the outdoor environment, there is an urgent need to manage these mosquito breeding sites. In order to elaborate more on the ecological landscape of mosquito breeding sites, the bacterial community structure and their interactions with physicochemical factors in mosquito larval habitats was characterised in Kwale County (Kenya), where malaria is endemic.

**Methods.:**

The physical characteristics and water physicochemical parameters of the habitats were determined and recorded. Water samples were also collected from the identified sites for total metagenomic DNA extraction in order to characterise the bacterial communities within the breeding sites.

**Results and Discussion.:**

Sites where mosquito larvae were found were described as positive and those without mosquito larvae as negative. Electrical conductivity, total dissolved solids, salinity and ammonia were lower in the rainy season than in the dry season, which also coincided with a high proportion of positive sites. Pseudomonadota was the most common phyla recovered in all samples followed by Bacteroidota and then Actinomycetota. The presence or absence of mosquito larvae in a potential proliferation site was not related to the bacterial community structure in the sampled sites, but was positively correlated with bacterial richness and evenness.

**Conclusion.:**

Generally, the presence of *Anopheles* mosquito larvae was found to be positively correlated with rainy season, bacterial richness and evenness, and negatively correlated with electrical conductivity, total dissolved solids, salinity and ammonia. The findings of this study have implications for predicting the potential of environmental water samples to become mosquito proliferation sites.

## Data Summary

The authors confirm that all supporting data, code and protocols have been provided within the article. All sequence data generated in this project was deposited in the National Centre for Biotechnology Information (NCBI BioProject ID: PRJNA953183; BioSample accession numbers: SAMN34109148, SAMN34109149, SAMN34109150, SAMN34109151, SAMN34109152, SAMN34109153, SAMN34109154). All physicochemical data, raw sequence data, taxonomic classification data and figures supporting this work have been deposited in the Microbiology Society’s data repository Figshare account [1].

## Introduction

Most studies have implicated *Anopheles* mosquitoes as the leading vectors of malaria parasites in Sub-Saharan Africa [2]. They are found both in urban and rural areas but with high populations in relatively wet regions near significantly large and permanent water bodies such as lakes and oceans [3, 4]. In regions where the vector population is high, malaria is endemic. These mosquitoes commonly oviposit in small, sunlit, semi-permanent and turbid water bodies like animal footprints, the edges of boreholes, puddles on the roadside formed by tires of vehicles and tracks, irrigation canals and other artificial water sources [5].

The choice of egg-laying sites by female *Anopheles* mosquitoes depends on the biotic and abiotic factors present in specific aquatic habitats. This, in turn, affects the abundance and distribution of their larvae [6], leading to varying levels of vector distribution and abundance in a specific region which in turn affects the spatio-temporal patterns of vector distribution and abundance within a given region [7]. Despite a lack of understanding of the key factors affecting the proliferation sites and the driving forces behind oviposition site preference, even for the most prominent malaria vectors [8], it has been suggested that mosquitoes lay their eggs in locations that provide optimal conditions for larval survival and growth such as stagnant water, warm temperature of between 24–27 °C, shaded areas, oxygen, nutrients, and a neutral pH, thus increasing the chances of success for their species [9].

Although a malaria vaccine is being developed, none has been rolled out yet for widespread use, and therefore prevention of transmission by mosquitoes remains the best option for preventing malaria infections. To date, vector control remains the most effective way to prevent malaria [10, 11]. Most vector control strategies have targeted the indoor host seeking behaviour of the mosquitoes which has succeeded to a large extent [12, 13]. Despite this remarkable success, elimination of malaria remains a big challenge since the malaria parasite is maintained by mosquitoes which oviposit, feed and rest in the outdoor environment [14]. Because of this setback together with the emergence of highly drug-resistant malaria parasites [15], there is an urgent need to focus on the management and control of oviposition sites seeking malaria vectors [16 ]. Furthermore, in malaria endemic countries like Kenya, efficient intervention and preventive protocols should be guided by knowledge of the abundance, distribution and characteristics of the proliferation sites of these vectors if malaria were to be effectively eliminated [17]. Environmental management is relatively simple and cost-effective compared to other mosquito control measures. Since mosquitoes are becoming resistant to insecticides, and constant use of insecticides can lead to further resistance, environmental management which reduces the use of insecticides will limit the chances of resistance development. Again, environmental management is an eco-friendly way of controlling mosquito populations, and therefore promotes environmental sustainability.

In order to effectively control mosquitoes, a comprehensive understanding of their larval ecology is essential. This includes examining the interplay between biotic and abiotic factors in breeding habitats, such as the types and preferences of breeding sites, the distribution and abundance of those sites, and the biological and physico-chemical conditions present [6]. Research has suggested that proper management of mosquito breeding habitats in sub-Saharan Africa could help reduce vector populations and curb malaria transmission [18]. By analysing the choice of oviposition sites and its impact on the distribution and abundance of malaria vector mosquitoes, we may be able to explain differences in malaria transmission intensity across different regions [19]. This information is valuable in the creation of integrated control strategies for *Anopheles* mosquitoes and health education programmes at the community level, aimed at lowering mosquito populations and reducing the risk of human-vector contact.

According to the 2020 World Health Organization (WHO) World Malaria Report, Kenya had 5.6 million confirmed cases of malaria in 2019, with the majority of cases occurring along coastal Kenya. In the coastal region, the prevalence of malaria varies depending on the specific location and time of the year. Generally, the risk of malaria transmission is higher in lowland areas, especially during the rainy season when there is an increase in mosquito breeding sites. However, due to the efforts of the Kenyan government and various international organisations, there has been a significant decline in malaria prevalence in the coastal region. For example, the prevalence of malaria in Mombasa County reduced from 27 % in 2016–16 % in 2019 according to Kenya Malaria Indicator Survey, 2020. Despite these initiatives, malaria continues to be a major cause of disease and death, particularly among young children, pregnant women, and those with compromised immune systems in coastal Kenya.

This challenge has been compounded by mosquito resistance to common insecticides which is becoming a major concern [20]. The emergence of drug resistance in mosquitoes as a result of excessive insecticide use, reduces the effectiveness of the insecticides, and this has made it difficult for researchers to find effective ways of controlling mosquito populations. It is advisable to employ approaches like integrated vector and environmental management, to adequately control mosquito populations and prevent the development of resistance to insecticides [21]. For example, the combination of insecticides and biological management strategies or modifying the environment to eliminate or reduce the breeding sites of mosquitoes.

At present, the knowledge about the impact of proliferation site distribution, biotic and abiotic factors on the distribution and density of malaria vectors in Kenya is scarce and inadequate to explain the patterns of adult mosquito distribution and abundance with certainty. This makes it difficult to implement effective malaria vector control strategies through the management of the larval forms [22–25]. Characterising the bacterial communities in mosquito breeding sites can help researchers to understand how mosquitoes survive, and how these bacterial communities interact with mosquitoes in these environments. For instance, some bacteria may be beneficial while others may be harmful to mosquitoes. By understanding how these communities function, researchers may be able to develop new strategies that specifically disrupt these interactions that mosquitoes rely on for survival. In response to this gap in knowledge, this study aims to characterise the total bacterial community structure and their interactions with physico-chemical ecological factors in mosquito breeding habitats in LungaLunga along the Kenyan coast, where malaria is widespread.

## Methods

### Study area

**Fig. 1.**
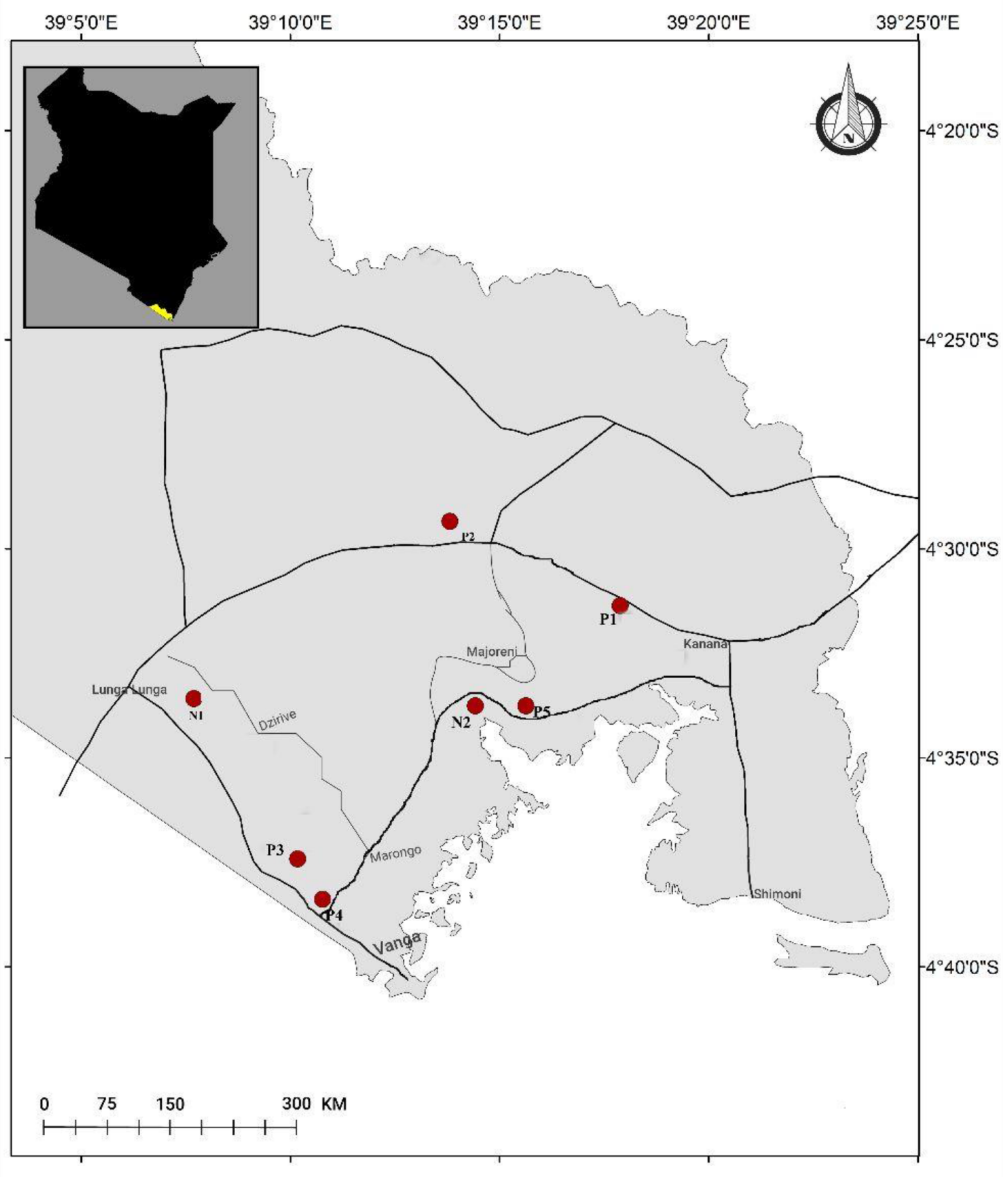
A map showing the sampling sites along three major roads within Lunga Lunga sub-county in Kwale county (Kenya). Samples were collected along the Ramisi-Lunga Lunga road between Kanana junction (coordinates: −4° 32' 21.822'', 39° 21' 59.281'') and the Umba river in Lunga Lunga town (coordinates: −4° 33' 16.678'', 39° 7' 33.121''), the road between Lunga Lunga town (coordinates: −4° 33' 16.679'', 39° 7' 33.121'') and Ngozi Girls Secondary School in Jego village (coordinates: −4° 35' 25.346'', 39° 9' 32.242'), and the road between Jego Village (coordinates: −4° 35' 25.346'', 39° 9' 32.242') and Kanana junction on Lunga Lunga-Ramisi road (coordinates: −4° 32' 21.822'', 39° 21' 59.281'').

The mosquito breeding sites were sampled along three major roads in Lunga Lunga sub-county, Kwale County, located along the South Coast of Kenya (as shown in Fig. 1). The selection of the sampling sites was based on the presence of larval habitats and their accessibility during the rainy season. Samples were collected along the Ramisi-Lunga Lunga road between Kanana junction (coordinates: −4° 32' 21.822'', 39° 21' 59.281'') and the Umba river in Lunga Lunga town (coordinates: −4° 33' 16.678'', 39° 7' 33.121''), the road between Lunga Lunga town (coordinates: −4° 33' 16.679'', 39° 7' 33.121'') and Ngozi Girls Secondary School in Jego village (coordinates: −4° 35' 25.346'', 39° 9' 32.242'), and the road between Jego Village (coordinates: −4° 35' 25.346'', 39° 9' 32.242') and Kanana junction on Lunga Lunga-Ramisi road (coordinates: −4° 32' 21.822'', 39° 21' 59.281'').

The region experiences two rainy seasons each year, between March to June and from October to November, with significant variations each year. For example, during the time of the study, there was no rainfall in October but the rainy season started towards the end of November until the end of December. Most of the residents in the region rely on small-scale farming and fishing to make a living. The area has a high prevalence of malaria among its local residents. Three species of malaria vectors, including *Anopheles arabiensis*, *Anopheles gambiae s.s*., and *Anopheles funestus*, have been previously identified in the region [26, 27 ].

### Study design and sample size

We conducted a cross-sectional study where 35 proliferation sites were sampled during the dry season (June to October, 2021 and January to April, 2022) and 30 sites during the rainy seasons (November to December, 202), according to the formula as described by Naing and others [28];

$$n=\frac{Z^{2}pqD}{d^{2}}$$

whereby *n*=required sample size, Z=standard normal variate which is 1.96, *P*=anticipated probability at 99 %, q=failure (1 p), D=design effect of control given a value of 2, and d=allowable error (0.05).

## Sample collection

### Identification of proliferation sites

Before the actual sample collection, each sampling site was accurately located using a GPS device (Garmin, Gpsmap 64, Garmin International Inc., Switzerland). The physical characteristics of the sites, including their natural or artificial nature, permanence, substrate type, depth, size, and vegetation, were recorded. Each potential mosquito breeding site was first visually inspected for the presence of larvae, and if larvae were not detected, a minimum of ten dips were made using a standard 350 ml dipper (BioQuip products, Rancho Dominguez, USA) to confirm the absence of larvae. A site was considered positive if at least one larva was found, and negative if no larvae were detected. The samples were collected from the selected mosquito breeding sites during both the dry and rainy seasons between June 2021 and April 2022. The sample collection was done between 7.00 am and 6.00 pm.

### Water samples collection

From each selected site, a single 500 ml and two 250 ml of water samples were collected using sterile plastic and glass bottles, respectively. The sampling bottles were first rinsed with the site water, which was carefully discarded before the sample was collected. Three controls were also included in the sample collection process. Nuclease-free water (500 ml) was used as a control by opening and uncapping the bottle during sampling. The 500 ml water samples were set aside for metagenome analysis, while the two 250 ml water samples were split as follows: one for nutrient analysis (nitrates, nitrites, ammonium, and phosphates) and the other for the determination of Biological Oxygen Demand (BOD). The sample for the BOD determination was wrapped in aluminium foil to keep out light and prevent photosynthetic activity, which could alter the concentration of oxygen in the bottles. Samples for nutrient analysis were kept at ambient temperature, while those for metagenome analysis and BOD determination were preserved in a cooler box with ice packs and transported to the laboratory immediately for processing.

### Mosquito proliferation sites water quality

Physico-chemical parameters, including water conductivity, temperature, total dissolved solids (TDS), dissolved oxygen (DO), pH, hardness (calcium and magnesium ions), and salinity, were measured *in situ* at each selected site using a YSI Professional Plus (Pro Plus) multi-parameter water meter (manufactured by YSI Inc., located in Yellow Springs, Ohio, USA). Three measurements were taken for each parameter. Turbidity was measured using a pre-calibrated AQUAfast AQ3010 turbidity meter (manufactured by Thermo Fisher Scientific, USA) following the manufacturer’s instructions.

The biological oxygen demand (BOD) of all the collected water samples was determined using the ManTech PC-BODTM analyser (located at Highway 6 North Guelph, Ontario N1H 6J2 Canada), which provides automated BOD analysis technology. The nutrients (nitrates, nitrites, ammonium, and phosphates) in the samples were analysed using the QuAAtro AutoAnalyser (manufactured by SEAL Analytical, located at Porvair Sciences Clywedog Road South, Wrexham Industrial Estate, Wrexham, United Kingdom), which employs a continuous segmented flow analysis (CFA/SFA) technique.

#### Mosquito larvae collection

The collection of mosquito larvae from the selected sites was performed using standard 350 ml larval dippers (BioQuip products, Rancho Dominguez, USA). To ensure adequate collection of larvae, several dips were made at each positive site, and all collected larvae were placed into 2 litre plastic containers. After collection, the larvae were immediately transported to the laboratory for further analysis and examination. The use of standard larval dippers and plastic containers ensured that the larvae were collected and transported in a safe and secure manner, minimising the risk of contamination and preserving their viability for further analysis.

### Morphological characterization of mosquito larvae

The collected mosquito larvae were filtered and placed in shallow plastic trays containing tap water. To provide proper nutrition and growth conditions for the larvae, 200 mg of powdery tetramin baby fish feed was added to the trays every morning. The water in the trays was changed every 3 days to ensure a clean and healthy environment for the larvae. Once the larvae pupated, the pupae were collected using a 5 ml plastic dropper and transferred to 500 ml plastic cups for the adult mosquitoes to emerge. The cups were covered with a fine cotton net and secured with a rubber band, with a small opening created in the centre for aspirating the emerging adult mosquitoes. This opening was covered with a piece of cotton wool to prevent any mosquitoes from escaping. Once the adult mosquitoes emerged, they were aspirated into 15 ml sterile vials using a standard mouth aspirator (Model 412) and stored in a refrigerator at 4 °C to allow the mosquitoes to die. The morphological features of the adult mosquitoes were then observed under a dissecting light microscope and identified based on morphological characters described in previously published keys [29]. *Anopheles* mosquitoes were identified up to species level while *Culex* mosquitoes were only identified up to genus level.

### 16S rRNA gene-based analysis

#### Sample preparation and total metagenomic DNA extraction

The thirty water samples collected were grouped into seven final samples based on the proximity of the sites and the presence or absence of mosquito larvae. Samples that were collected from sites where mosquito larvae were observed were labelled as P1, P2, P3, P4, and P5, while those without larvae were labelled as N1 and N2. The samples were collected from different regions along the roads, where P1 and P2 were obtained from sites located between Kanana Junction (coordinates: −4° 32' 21.822'', 39° 21' 59.281'') and River Umba in Lungalunga (coordinates: −4° 33' 16.678'', 39° 7' 33.121''), N1 and P3 from sites located between River Umba (coordinates: −4° 33' 16.679'', 39° 7' 33.121'') and Ngozi Girls Secondary School in Jego village (coordinates: −4° 35' 25.346'', 39° 9' 32.242'), and P4, N2, and P5 were collected from sites between Jego village (coordinates: −4° 35' 25.346'', 39° 9' 32.242') and Kanana Junction (coordinates: −4° 32' 21.822'', 39° 21' 59.281'') as shown in Fig. 1.

The preparation of the water samples for the extraction of total metagenomic DNA was performed as described before [30 ]. One litre of each of the final samples was filtered through sterile 0.22 µm filter membranes (Merck Millipore, Burlington, MA) to trap bacterial cells. The filter membranes were aseptically removed from the filtration apparatus and cut into four pieces using a sterile pair of forceps and scissors. The pieces were then placed along the bottom of a 50 ml sterile conical tube with the upper surface of the filter facing the centre of the tube. Thirty millilitres of extraction buffer were added to the tube. The trapped biomass was washed off the filters by vortexing the tubes vigorously, and the cell suspension was transferred to a clean microcentrifuge tube. The tube was incubated in a heating block at 65 °C for 30 min, with gentle vortexing after every 10 min. After the incubation period, the tube was allowed to cool to room temperature, and an equal amount of chloroform: isoamyl alcohol (24 : 1 v/v) was added and mixed by gentle inversion. The mixture was then centrifuged at 13 200 r.p.m. for 5 min at room temperature, and the supernatant was transferred to a new 50 ml tube. Total genomic DNA was then precipitated, cleaned, and resuspended in nuclease-free water. The concentration and purity of the extracted DNA were assessed using 1 % agarose gel electrophoresis [31 ] and a NanoDrop spectrophotometer [32 ], then stored at −40 °C.

#### Next generation sequencing

In this study, the 16S rRNA gene was targeted and amplified using the primers F27 ‘AGRGTTYGATYMTGGCTCAG’ and R1492 ‘RGYTACCTTGTTACGACTT’ [33]. The annealing temperature was optimised around 55 °C. An initial denaturation step at 95 °C for 5 min, followed by 35 cycles of amplification at 95 °C for 30 s, annealing temperature for 30 s, and 72 °C for 60 s, a final extension step at 72 °C for 10 min. The amplified product was then sequenced on the PacBio Sequel platform using PacBio Barcoded M13 Primers for Multiplex SMRT Sequencing. A positive control sample containing 17 known bacterial isolates was used as a mock to test the sequencing and analysis pipelines.

#### Metataxonomics

PacBio sequences obtained were processed and visualised using the RS_ReadsOfInsert protocol in the SMRT Analysis software version 2.3 to obtain demultiplexed consensus sequences with a minimum of three full passes. The resulting sequence data were processed using the Divisive Amplicon Denoising Algorithm2 (DADA2) pipeline [34] in R version 4.2.1, R Core Team (2022) as follows. First, the F27 and R1492 primers were removed from the raw sequences and the quality of the reads was inspected. The sequences were then filtered using the parameters; minQ=2, minLen=500, maxLen=1600, maxN=0, rm. phix=FALSE, maxEE=2; minQ=2 sets the minimum quality score for each base in the sequence. A quality score of 2 represents a 1 % error probability, meaning that a base with a quality score of 2 may be incorrect only 1 % of the time. minLen=500 sets the minimum length of the reads used in the analysis. Sequences shorter than 500 bases were excluded from the analysis. maxLen=1600 sets the maximum length of the reads used in the analysis. Sequences longer than 1600 bases were excluded from the analysis. maxN=0 specifies the maximum number of ambiguous bases (N) allowed in each sequence. A value of 0 means that no ambiguous bases were allowed in the sequences. rm.phix=FALSE specifies whether to remove reads derived from the phix174 genome. A value of ‘FALSE’ means that phix174 genome reads were not removed. maxEE=2. sets the maximum number of expected errors allowed in each sequence. The expected error rate is calculated from the quality scores of the bases. A value of 2 means that sequences with an error rate greater than two were excluded from the analysis.

Taxonomic classification of the filtered reads was then assigned to the species level using kraken2 pipeline [35], implementing the bacteria refseq database of the NCBI (NCBI Bacterial RefSeq Database. http://ftp.ncbi.nlm.nih.gov/refseq/release/bacteria/bacteria.1.1.genomic.fna.gz. Accessed 30 May 2023). To assess the reliability of the sampling depth, the OTU tables were rarefied and the precision of the rarefaction curves was estimated using the bootstrapping method [36]. Alpha diversity measures were calculated using *vegan* package version 2.6–2 [37] in R from the number of OTUs. Shannon diversity estimate based on species richness and evenness emphasising more on species richness and Simpson diversity index based on species richness and evenness putting more weight on species evenness [38–40].

All statistical data analysis was performed in XLSTAT [41] and R statistical programme version 4.2.1 (R Core Team, 2022). Physical characteristics of positive and negative sites were represented in percentages and compared using the Z-test. Physicochemical data was summarised using mean and standard deviation. Student’s t-test and Permutational Multivariate Analysis of Variance (PERMANOVA) were used to compare physicochemical parameters between the positive and negative sites at 95 % confidence interval [42–45]. The Principal components analysis (PCA) was conducted to identify the relationships between different parameters and the sites. To test whether there was any significant effect associated with the physicochemical parameters on the alpha diversity of bacteria, non-parametric Kruskal-Wallis test was performed [46].

## Results

### Distribution of mosquito sites

**Fig. 2.**
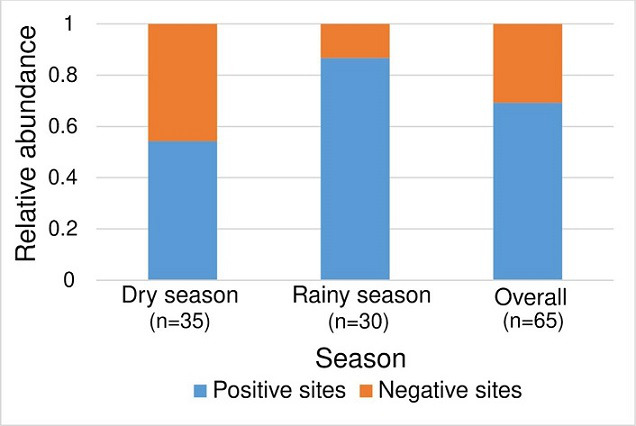
Abundance of mosquito larval habitats, During the dry season (June to October, 2021 and January to April, 2022), the number of positive sites was comparable to that of negative sites (*P*=0.321, 95 % CI: 0.068, 0.228). However, during the rainy season (November to December, 2021), there were more positive sites than negative ones (*P*<0.0001, 95 % CI: 0.637, 0.843).

We sampled 35 sites during the dry season (June to October, 2021 and January to April, 2022) and 30 during the wet season (November to December, 2021), and evaluated them for the presence (positive) or absence (negative) of mosquito larvae as described in Fig. 2. During the dry season, 19 sites (54.28 %) were positive and 16 (45.72 %) were negative, indicating that the number of positive and negative sites were not significantly different (*P*=0.321, 95 % CI: 0.068, 0.228). However, in the rainy season, we found that 26 (86.67 %) out of 30 sampled sites were positive and only four (13.33 %) were negative for mosquito larvae (*P*<0.0001, 95 % CI: 0.637, 0.843). Overall, the positive sites for the presence of mosquito larvae were 69.23 % while the negative sites were 30.77 % (*P*<0.0001, 95 % CI: 0.242, 0.518). The proportion of positive sites was also significantly higher in the rainy season (86.67 %) than in the dry season (*P*<0.0001, 95 % CI: 0.158, 0.442).

### Abundance of mosquito larvae in the positive sites

**Fig. 3.**
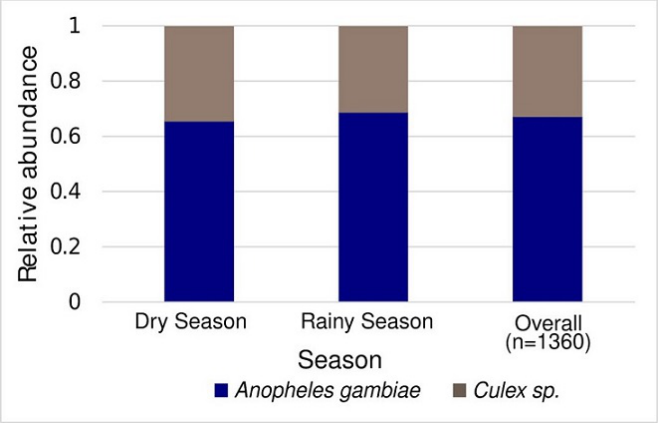
Abundance of mosquito species in larval habitats (*N*=1360), The majority of the sampled sites were found to contain *Anopheles gambiae* larvae, with only a few containing *Culex* mosquitoes (P ˂ 0.0001, 95 % CI: 0.263, 0.567).

We collected 1360 mosquito larvae from the positive sites and reared them into adult mosquitoes, which were then identified as *Anopheles gambiae* or *Culex* sp., with the abundance of *Anopheles gambiae* being 68.34 % (P ˂ 0.0001, 95 % CI: 0.221, 0.499) and 71.32 % (P ˂ 0.0001, 95 % CI: 0.306, 0.574), during the dry and rainy seasons respectively (Fig. 3). The average percentage of *Anopheles gambiae* across both seasons was 69.83 % (P ˂ 0.0001, 95 % CI: 0.263, 0.567).

### Occurrence of mosquito larvae in the sites

**Fig. 4.**
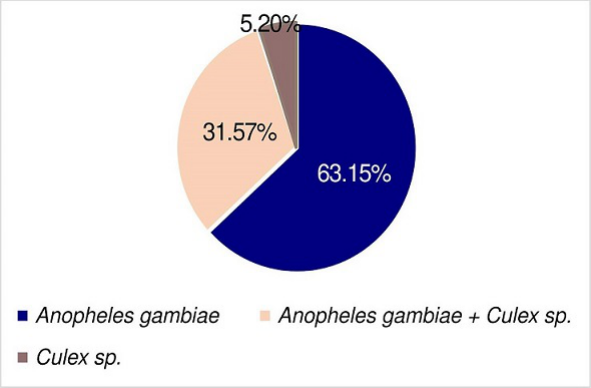
Abundance of mosquito larvae in the positive sites (*N*=1360), 63.15 % of the positive sites had *Anopheles gambiae* larvae only, 5.20 % had *Culex* sp. larvae only, and 31.65 % had both *Anopheles gambiae* and Culex sp. larvae (X^2^=5.991, df=2, *P*˂0.0001).

We found that 63.15 % of the positive sites had *An. gambiae* larvae only, 5.20 % had *Culex* sp. larvae only, and 31.65 % had both *An. gambiae* and *Culex* sp. larvae (X^2^=5.991, df=2, *P*<0.0001) as shown in Fig. 4. This suggests that during the study period, more habitats were suitable for the breeding of *An. gambiae* compared to other species.

### Physical characteristics of proliferation sites

**Fig. 5.**
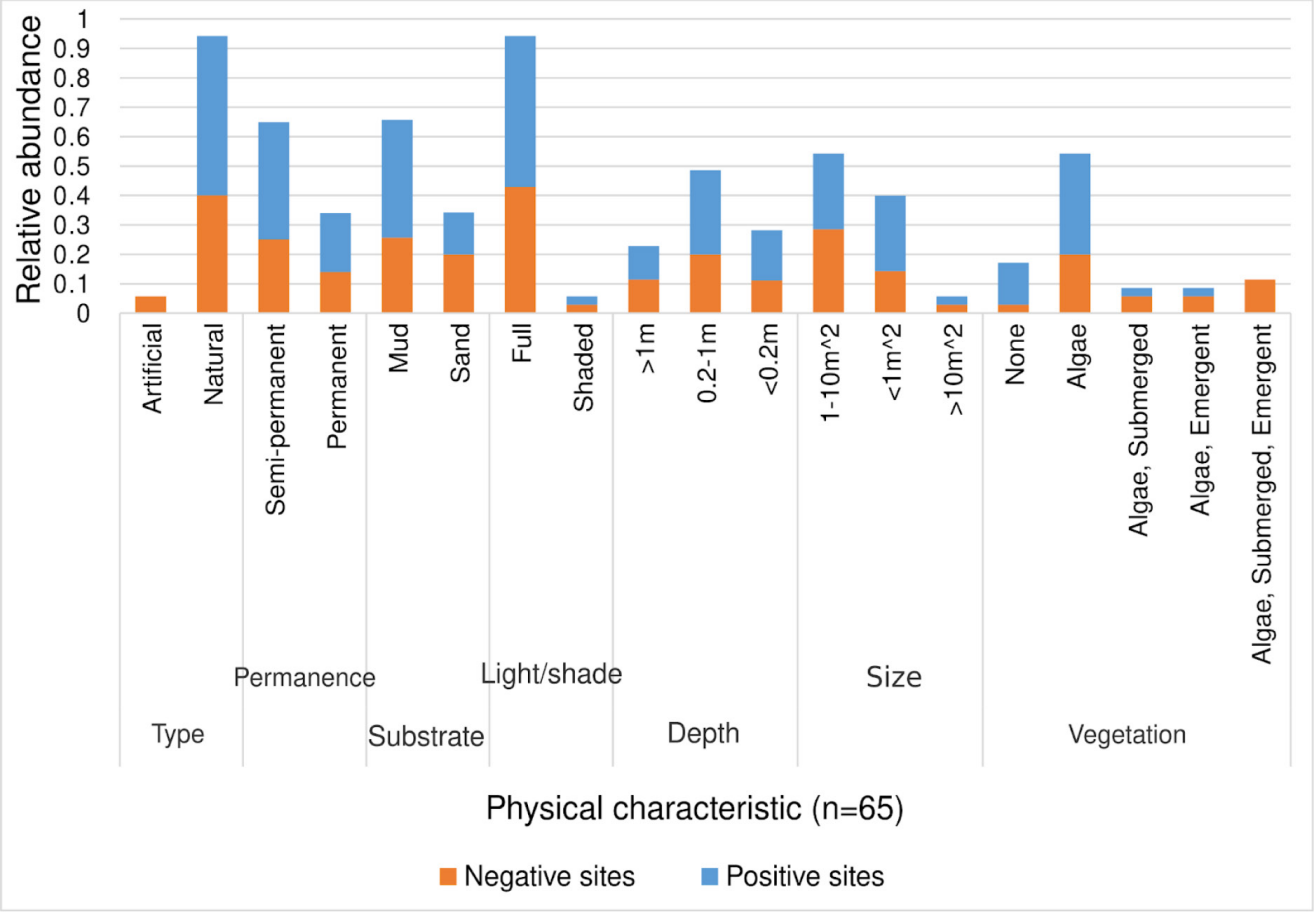
Physical characteristics of larval habitat, most of the sites were natural (*P*<0.0001, 95 % CI: 0.8295, 0.9704), semi-permanent (*P*<0.0001, 95 % CI: 0.158, 0.442), had mud substrate (*P*=0.006, 95 % CI: 0.179, 0.461), were fully exposed to sunlight *P*<0.0001, 95 % CI: 0.8295, 0.9704), had a shallow depth of less than 1 m (*P*<0.0001, 95 % CI: 0.413, 0.667) with an area of less than 10 m^2^ (*P*<0.0001, 95 % CI: 0.8295, 0.9704), and had algae as the main vegetation (*P*<0.0001, 95 % CI: 0.144, 0.458).

These were determined based on 35 sites sampled during the dry season and 30 sampled during the rainy season. Most of the sites were natural habitats (94.28 %) while only 5.71 % were artificial, such as man-made dams and road culverts (*P*<0.0001, 95 % CI: 0.8295, 0.9704). Natural habitats included marshy areas, shallow rivers, roadside pools, and animal hoof-prints. We found 65.71 % of the sites had mud substrates while 34.28 % had sand (*P*=0.006, 95 % CI: 0.179, 0.461). In terms of permanence, 65.7 % of the sites were semi-permanent, while the rest were permanent (*P*<0.0001, 95 % CI: 0.158, 0.442). Most of the sites were fully exposed to sunlight (94.28 %, *P*<0.0001, 95 % CI: 0.8295, 0.9704) and had a shallow depth of less than 1 m (77.14 %, *P*<0.0001, 95 % CI: 0.413, 0.667) with an average size of less than 10 m^2^ (94.28 %, *P*<0.0001, 95 % CI: 0.8295, 0.9704). In terms of vegetation, 82.86 % of the habitats had some form of vegetation while 17.14 % had no vegetation at all (*P*<0.0001, 95 % CI: 0.546, 0.774). The habitats were grouped into four categories based on the type of vegetation present. The majority of the habitats had only algae (54.28 %, *P*<0.0001, 95 % CI: 0.144, 0.458). Some habitats had a combination of algae, submerged, and emergent vegetation (11.42 %), others had algae and emergent vegetation (8.57 %), and a few had algae with only emergent vegetation (8.57 %). Information on the physical characteristics of the sites is presented in Fig. 5.

### Physicochemical parameters of the sites

Although mean temperature at the positive sites was significantly lower than that in the negative sites during the dry season (t=1.729, df=19, *P*=0.0416), there was no significant difference in temperature between positive and negative sites during the rainy season (Table 1). None of the twelve physicochemical parameters studied differed significantly between positive and negative sites in both seasons (R2=0.1180, df=1, *P*=0.106). A pairwise comparison of the individual physicochemical parameters using the Student’s t-test between the dry and rainy seasons showed that salinity (t=1.692, df=33, *P*=0.01104), electrical conductivity (t=1.689, df=33, *P*=0.01617), total dissolved solids (t=1.690, df=33, *P*=0.01204), and ammonia (t=1.675, df=33, *P*=0.00029) were significantly lower during the rainy season compared to the dry season, while the other variables were not significantly different. The mean, standard deviations, and *p*-values for the Student’s t-test of the physicochemical parameters between positive and negative sites evaluated during the dry and rainy seasons are summarised in Table 2.

**Table 1.** Summary of physicochemical parameters

Season		Temp (^0^°C)	pH	DO (mg l^−1^)	BOD_5_ (mg l^−1^)	Salinity (ppt)	E.C (us m^−1^)	TDS (mg l^−1^)	TUR (NTS)	NO_3_^-^ (umol l^−1^)	NO_2_^-^ (umol l^−1^)	NH_3_ (umol l^−1^)	PO_4_^3 -^ (umol l^−1^)
**Dry season**	**Positive sites (X̅**) **Standard deviation**	30.82 ±2.37	8.62 ±0.69	7.52 ±5.0	5.347 ±5.58	2.19 ±1.73	4481 ±3398	2634 ±2012	386.2 ±311	24.9 ±12.9	6.02 ±3.25	20.25 ±12.4	3.21 ±3.03
	**Negative sites (X̅**) **Standard deviation**	32.57 ±1.93	8.26 ±0.81	5.74 ±3.2	2.708 ±2.14	3.058 ±5.2713	5363 ±893	3060 ±5136	319 ±417	21.71 ±12.1	4.98 ±3.74	27.03 ±12.7	4.65 ±4.3
	***P*-value**	0.041^✱^	0.206	0.27	0.115	0.5410	0.7183	0.7625	0.622	0.495	0.423	0.153	0.296
**Rainy season**	**Positive sites (X̅**) **Standard deviation**	32.14 ±1.28	8.47 ±0.55	5.88 ±2.0	2.8 ±1.64	0.67 ±1.1	1685.7 ±2228	898.51 ±1174	472.0 ±373	23.97 ±7.43	4.59 ±3.17	12.87 ±9.55	3.73 ±3.41
	**Negative Sites (X̅**) **Standard deviation**	31.58 ±2.52	8.167 ±0.33	5.59 ±2.2	2.232 ±1.67	0.36 ±0.58	1414 ±1898	723.01 ±1221	453.5 ±101	25.21 ±4.83	3.9 ±1.36	12.37 ±1.64	2.56 ±1.41
	***P*-value**	0.691	0.172	0.81	0.561	0.4175	0.8070	0.8009	0.835	0.677	0.466	0.803	0.249

Temp – Temperature, DO – Dissolved oxygen, BOD5 – Biological oxygen demand after 5 days incubation, E.C – Electrical conductivity, TDS – Total dissolved solids, TUR – Turbidity, NH_3_ - Ammonia, a summary of the physicochemical parameters between positive and negative sites evaluated during the dry and wet seasons shows that during the dry season, the mean temperature for positive sites was significantly lower than that of negative sites (t=1.729, df=19, ^✱^ *P*=0.0416), but this was not the case during the rainy season (t=1.89, df=19, *P*=0.691).

**Table 2.** Comparison of physicochemical parameters in the sites between dry and rainy seasons

Parameters (*N*=30)		Temp (°C)	PH (mg l^−1^)	DO (mg l^−1^)	BOD_5_ (mg l^−1^)	Salinity (ppt)	E.C (us m^−1^)	TDS (mg l^−1^)	TUR (NTS)	NO_3_^-^ (umol l^−1^)	NO_2_^-^ (umol l^−1^)	NH_3_ (umol l^−1^)	PO_4_^3 -^ (umol l^−1^)
**Dry season**	Mean SD	31.74±2.27	8.431±0.76	6.57±4.2	3.94±4.26	2.653±3.989	4951.8±6831	2861.9±3938	350.7±366	23.21±12.4	5.4685±3.5	23.87±12.8)	3.97±3.79
**Rainy season**	Mean SD	32.06±1.44	8.43±0.52	5.84±2.0	2.72±1.63	0.628±1.04	1650±2162	875.86±1161	469.6±349	24.14±7.1	4.5±2.99	12.81±8.91	3.58±3.23
**Overall**	Mean	31.9	8.433	6.20	3.32	1.624	3274.1	1852.6	411.1	23.67	4.98	18.25	3.776
	SD	±1.89	±0.65	±3.29	±3.23	±3.04	±5259	±3228	±360	±10.0	±3.262	±12.28	±3.49
***P*-value**		0.511	0.986	0.39	0.152	0.01104	0.0161^✱^	0.012^✱^	0.200	0.722	0.254	0.0003^✱^	0.6638

Temp – Temperature, DO – Dissolved oxygen, BOD5 – Biological oxygen demand after 5 days incubation, E.C – electrical conductivity, TDS – Total dissolved solids, TUR – Turbidity, NH_3_ – Ammonia, A pairwise comparison of the individual physicochemical parameters using the Student’s t-test between the dry and rainy seasons showed that salinity (t=1.692, df=33, *P*=0.01104), electrical conductivity (t=1.689, df=33, ^✱^ *P*=0.01617), total dissolved solids (t=1.690, df=33, ^✱^ *P*=0.01204), and ammonia (t=1.675, df=33, ^✱^ *P*=0.00029) were significantly lower during the rainy season compared to the dry season, while the other variables were not significantly different.

### Metataxonomic analysis of bacteria

### Sequencing reads quality control

Sequencing of the full 16S rRNA generated 203 934 reads from seven environmental samples with an average of 29 133 reads per sample (Table 3). The average length of the reads was approximately 1450 base pairs, which aligns with the expected full length of the 16S rRNA gene. After applying various quality control measures such as trimming, filtering, and denoising, we were left with 104 040 reads.

**Table 3.** Sequence reads distribution per sample

Sample	CCS	Primers	After QC
P1	27 250	22 977	13 206
P2	29 589	25 225	14 719
N1	33 903	29 121	19 033
P3	24 670	21 707	11 806
P4	37 548	32 977	21 498
N2	12 822	10 900	3878
P5	38 152	34 082	19 900
Total	203 934	176 989	104 040
Mean	29 133	25 284	14 862
±SD	±8799	±8501	±6049

CCS – Circular consensus sequence, QC - Quality control, CCS represents the number of raw reads, while after QC (quality control) represents the number of reads remaining after performing quality control.

### Taxonomic classification of the filtered reads

**Fig. 6.**
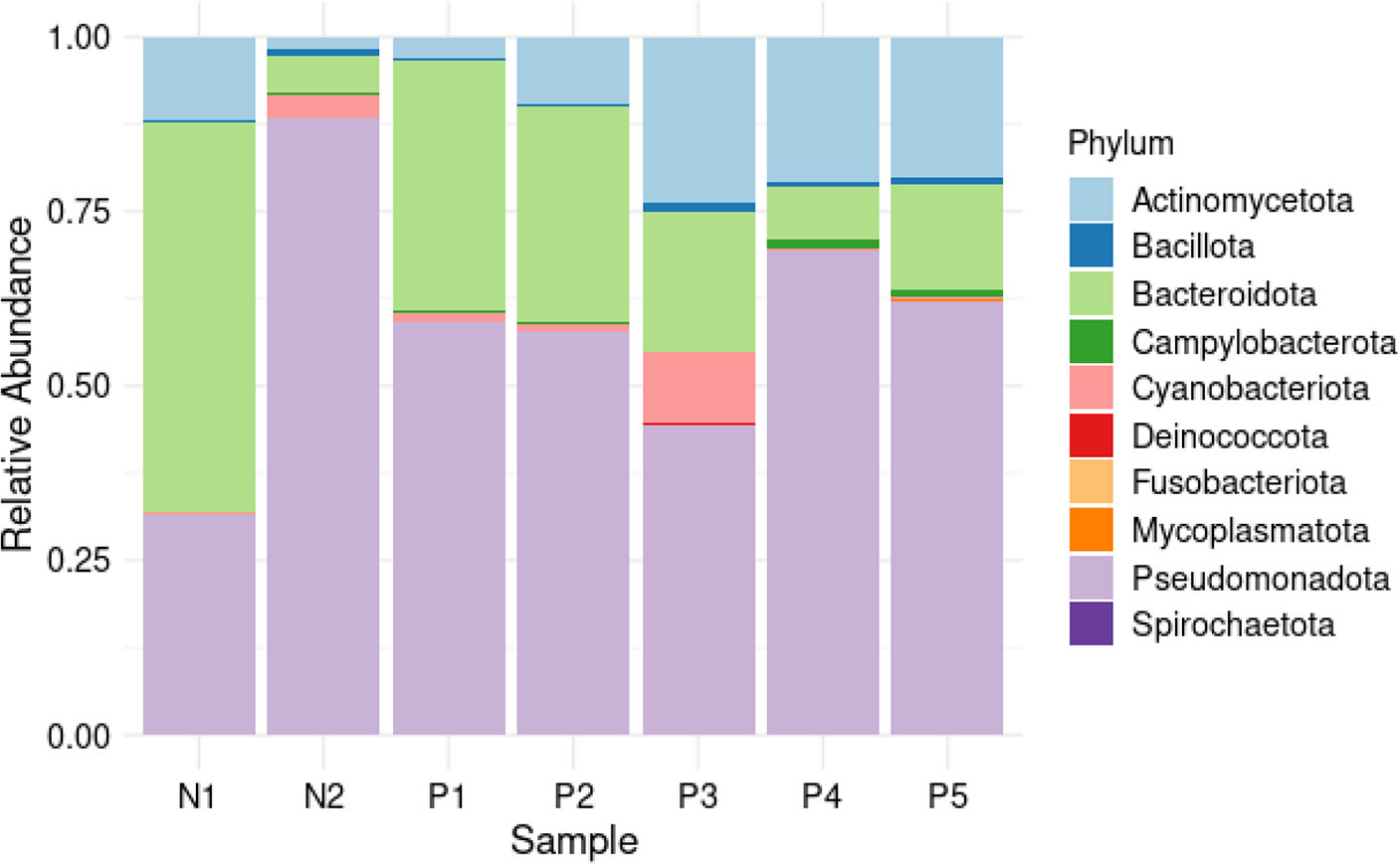
Phylum distribution. The stacked barplot depicts the relative abundance of bacterial phyla in the study samples. The negative group, represented by samples N1 and N2, and the positive group, represented by samples P1 to P5, are differentiated. Each sample is represented by a bar, and the height of the bar corresponds to the percentage of abundance. The colours used in the plot represent different bacterial phyla, providing insight into the composition of the microbial community across the samples.

**Fig. 7.**
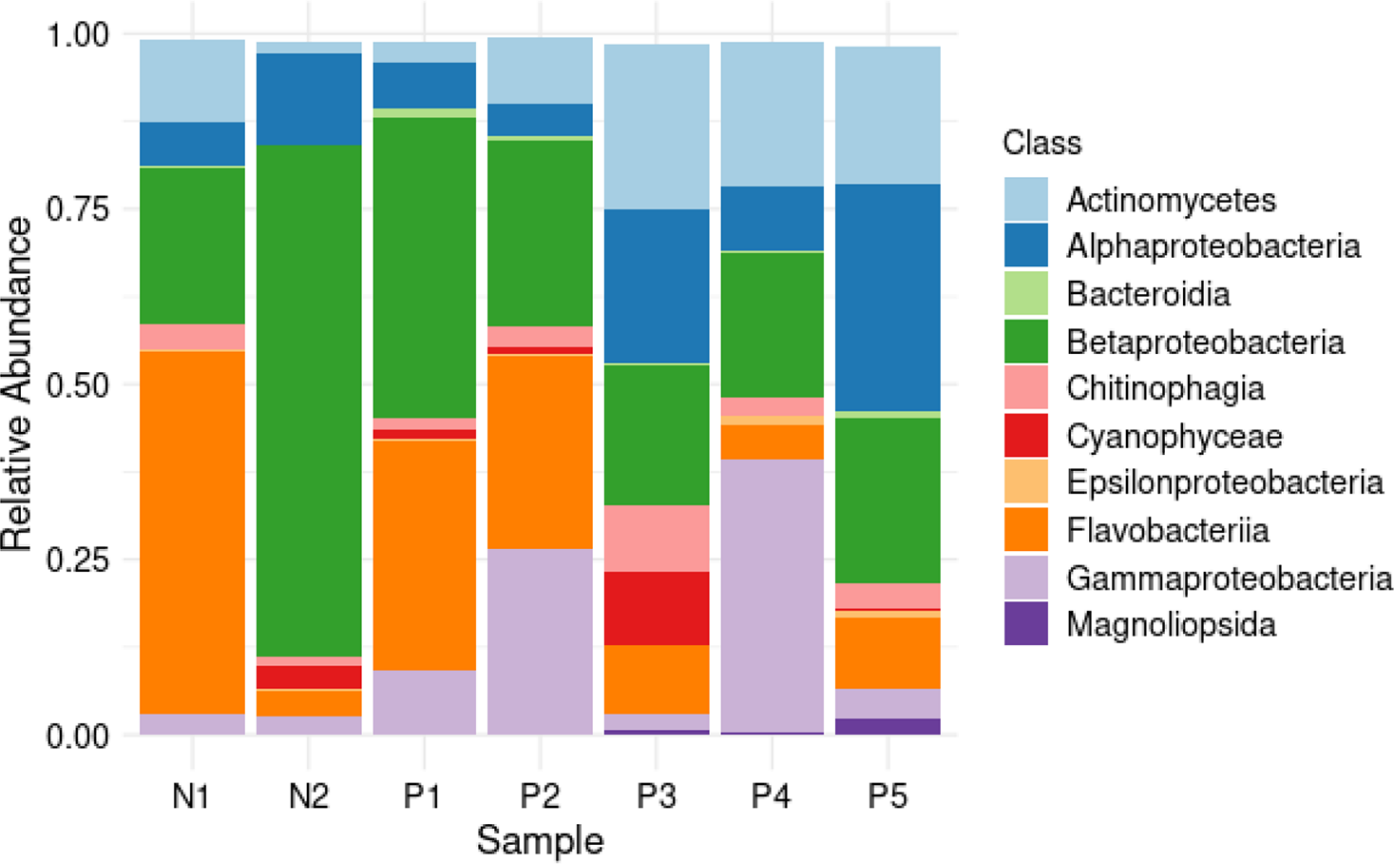
Class distribution. Stacked barplot showing top ten bacterial classes' relative abundance in negative (**n1, n2**) and positive (**p1-p5**) samples.

**Fig. 8.**
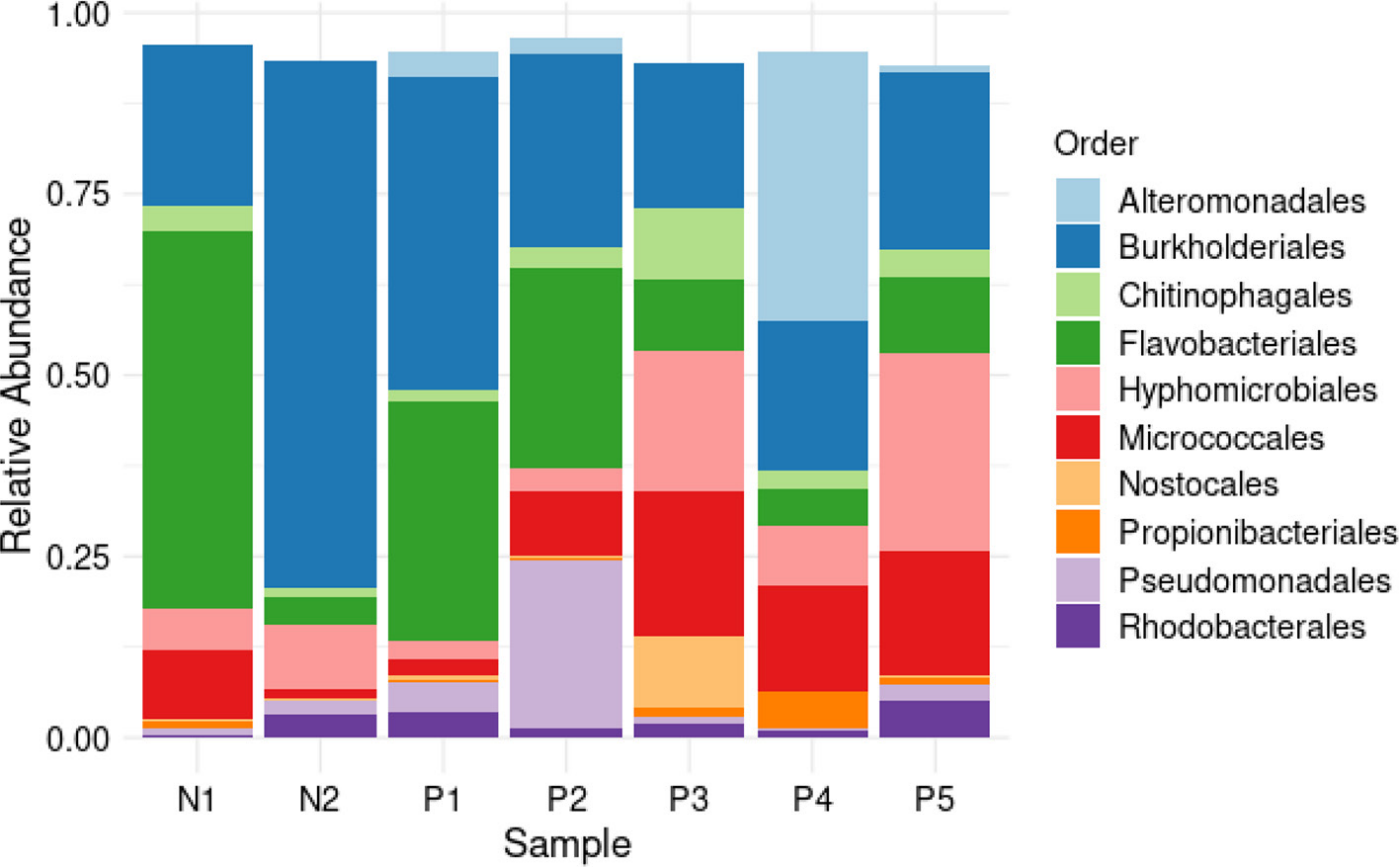
Order distribution. Stacked barplot showing top ten bacterial order’s relative abundance in negative (**n1, n2**) and positive (**p1-p5**) samples.

**Fig. 9.**
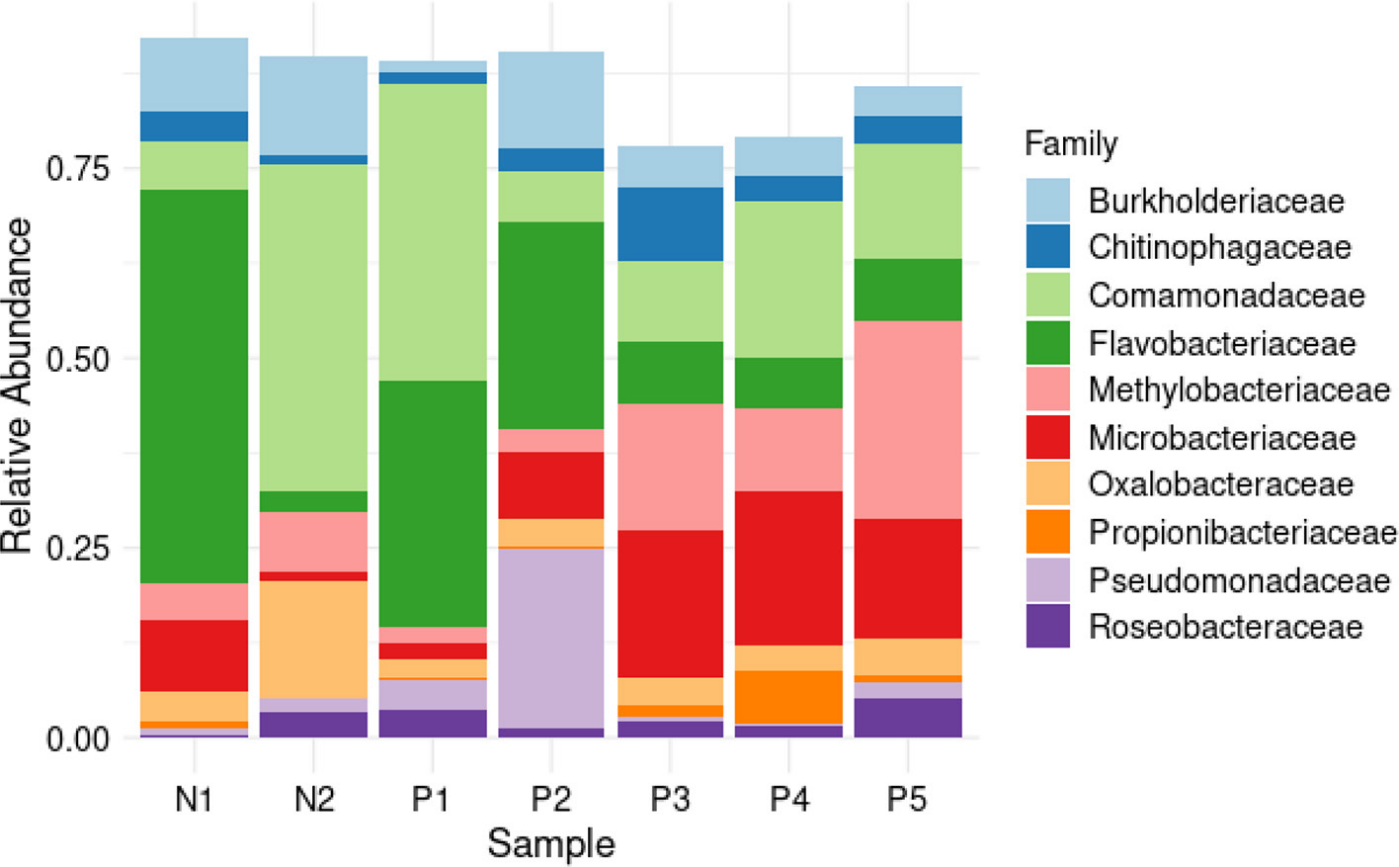
Family distribution. Stacked barplot showing top ten bacterial families' relative abundance in negative (**n1, n2**) and positive (**p1-p5**) samples.

**Fig. 10.**
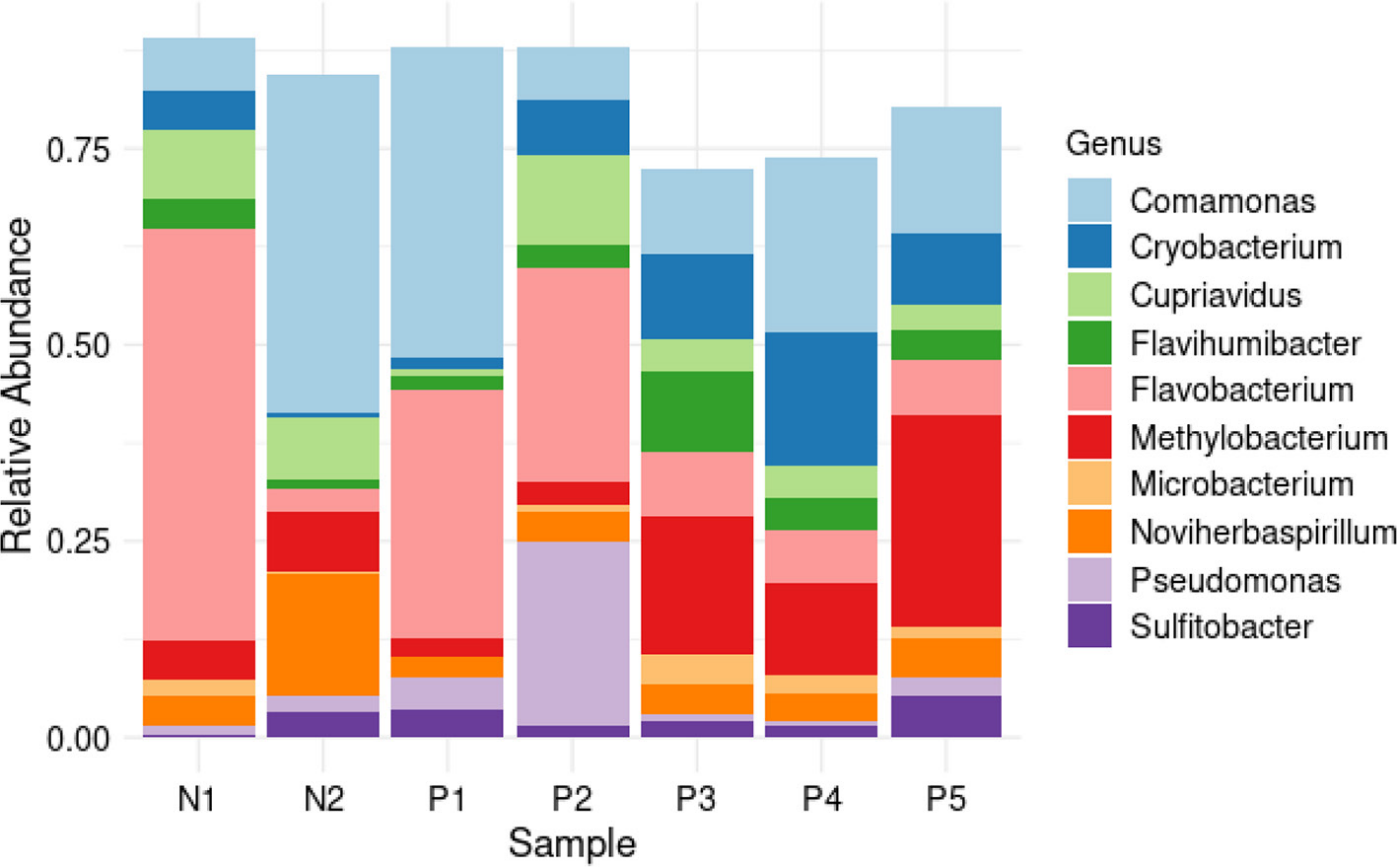
Genus distribution. Stacked barplot showing top ten bacterial genera relative abundance in negative (**n1, n2**) and positive (**p1-p5**) samples.

**Fig. 11.**
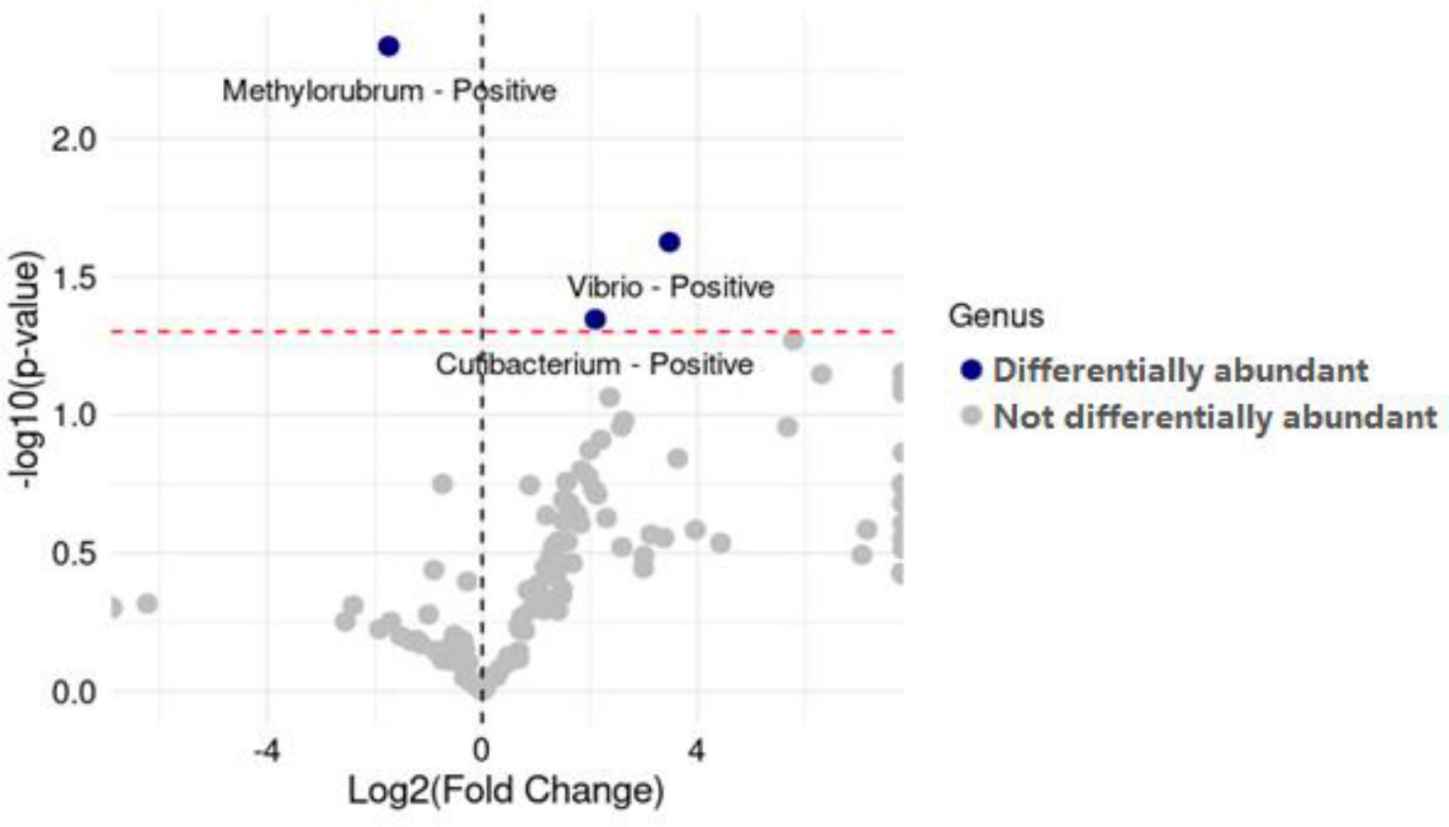
Differential abundance analysis of bacterial genera. The figure shows bacterial genera whose abundance was significantly different between the positive and negative sites. Each point represents a genus, with the x-axis representing the log2 fold change in abundance between the two groups, and the y-axis representing the -log10(*p*-value) indicating the significance of differential abundance. Points coloured in navy indicate genera that are differentially abundant, while points coloured in grey indicate genera that are not differentially abundant between positive and negative sites. The dashed vertical line represents the threshold for no fold change, and the dashed horizontal line represents the threshold for statistical significance. The labels indicate the genera names along with their corresponding group (Positive or Negative).

The ASVs were taxonomically assigned to 10 phyla, 40 classes, 87 orders, 129 families, 188 genera and 257 species. The most commonly detected phylum was Pseudomonadota, which accounted for 56 % of the total reads, followed by Bacteroidota (26%) and Actinomycetota (15%). The other seven phyla accounted for 3 % of all the phyla detected in all samples (Fig. 6). At the class level, Betaproteobacteria, Flavobacteriia, Gammaproteobacteria, and Actinomycetes were the most abundant (Fig. 7). The most common orders were Burkholderiales, Flavobacteriales, Micrococcales, Hyphomicrobiales, and Alteromonadales (Fig. 8). Out of all the families identified, Flavobacteriaceae, Comamonadaceae, Microbacteriaceae, Methylobacteriaceae, and Burkholderiaceae were the most abundant (Fig. 9). For the 188 genera, *Flavobacterium, Comamonas, Methylobacterium, Cryobacterium, Cupriavidus,* and *Pseudomonas* were the most detected (Fig. 10). Further, differential abundance analysis revealed significant differences in the abundance of bacterial genera between the positive and negative sites. Specifically, the genus *Vibrio* and *Cutibacterium* were found to be significantly more abundant in the positive sites compared to the negative sites (*P*<0.05), indicating a potential association with positive environmental conditions. On the other hand, the genus *Methylorubum* exhibited lower abundance in the positive sites compared to the negative sites (*P*<0.05). These findings suggest that *Vibrio* and *Cutibacterium* may play a role in positive site conditions, while *Methylorubum* may be more prevalent in the negative site environment. The volcano plot in Fig. 11 visually represents these differential abundance patterns, with points coloured in navy blue indicating differentially abundant genera and points coloured in grey representing genera with no significant differential abundance.

### Diversity of bacterial communities

**Fig. 12.**
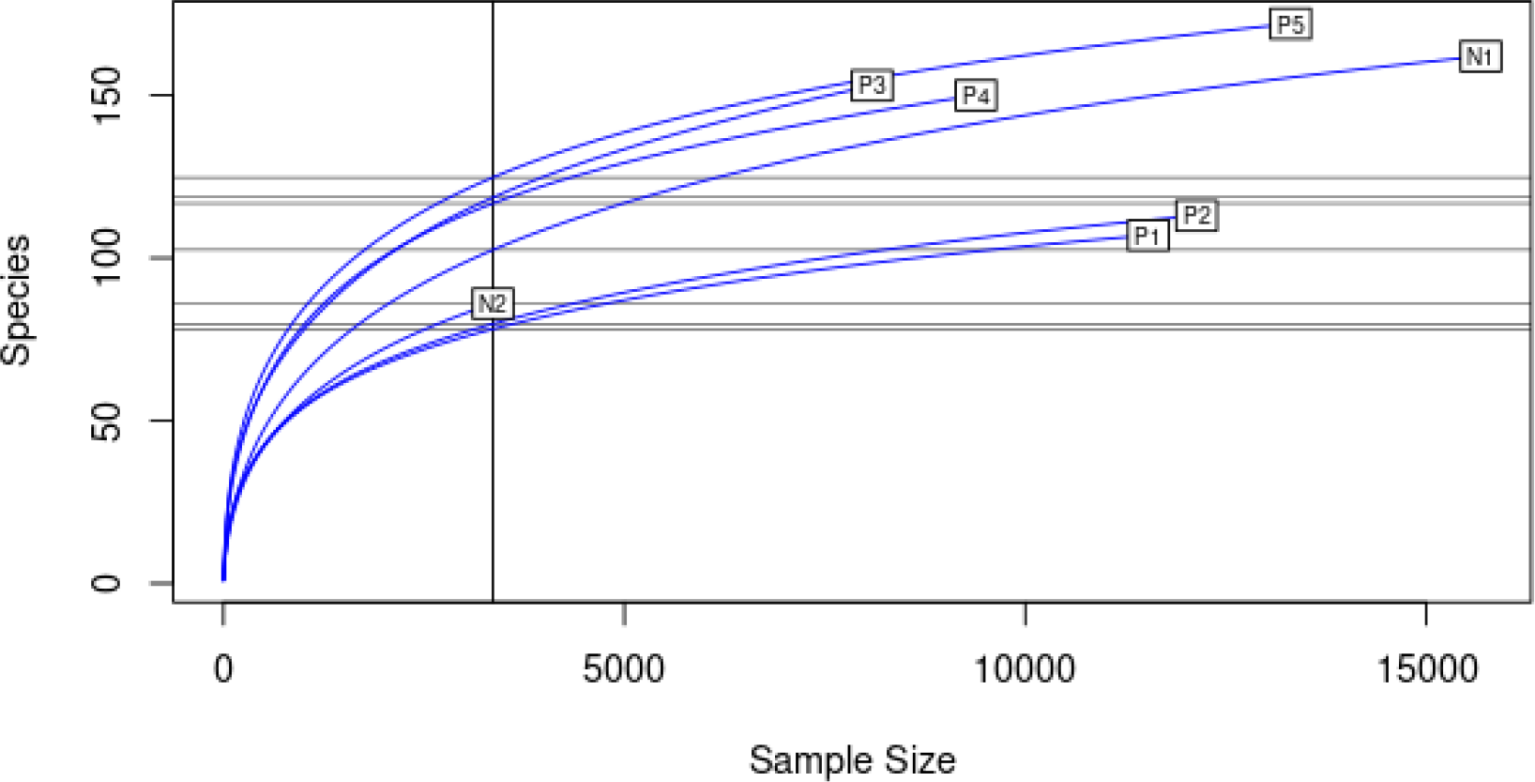
Rarefaction curve: exploring bacteria diversity variation cross samples with N2 as the minimum sample size.

**Fig. 13.**
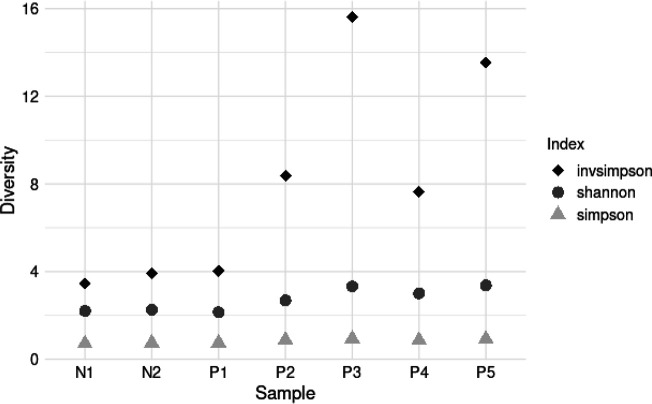
Alpha diversity indices comparing sites with and without mosquito larvae. The samples are divided into two groups: negative (N1-2), which denotes sites where mosquito larvae were not detected, and positive (P1-5), representing sites where mosquito larvae were found.

**Fig. 14.**
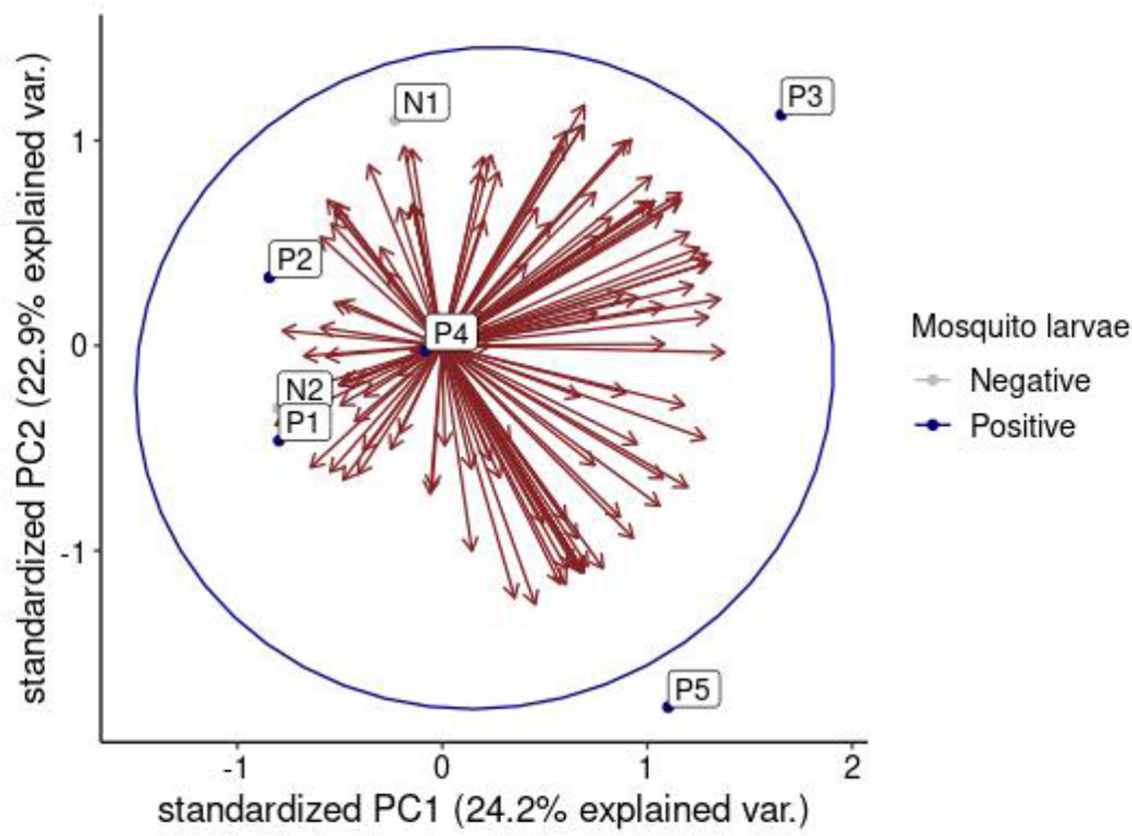
Principal Component Analysis (PCA). The PCA plot shows the relationship between samples and the bacterial species distribution. N1 and N2 samples represent samples from sites without mosquito larvae while samples P1–P5 represent samples from sites with mosquito larvae.

A rarefaction plot was used to compare biodiversity across different samples. Sample N2, which had the lowest species count, was used to determine the minimum sample size for downstream analysis. Rarefaction allowed for standardising the comparison of biodiversity by estimating species richness or diversity at the same level of sampling effort, irrespective of sample size (Fig. 12). Several alpha diversity indices for the positive and negative samples were also computed and compared as shown in (Fig. 13). All the alpha diversity indices were highest in sample P3 and lowest in sample N1 and they were highly variable between the sites, an indication that the bacterial community richness, evenness and abundance were not similar between sites (F=2.928, df=8, *P*=0.032). However, the physicochemical parameters tested had no effect on the diversity indices of bacterial communities between sites (Kruskal-Wallis chi-squared=6, df=6, *P*=0.4232). Samples taken from positive sites were noted to have higher alpha diversity indices in comparison to those from negative sites and therefore indicating that the presence of mosquito larvae was correlated with high bacterial richness and evenness (R^2^=9.822, df=1, *P*=0.00197). For beta diversity analysis, the positive and negative samples did not present any specific clustering pattern (Fig. 14), which is further supported by the statistical analysis (R^2^=0.18157, df=1, *P*=0.353). This suggests that the presence or absence of the mosquito larvae was not correlated with any particular bacterial community structure.

## Discussion

### Mosquitoes proliferation sites

During the dry season (June to October, 2021 and January to April, 2022), the number of positive sites was comparable to that of negative sites. However, during the rainy season (November to December, 2021), there were more positive sites than negative ones. This result indicates that the rainy season’s rainfall patterns can impact the reproduction and distribution of mosquitoes in the environment. The increased humidity and availability of water during the rainy season create favourable conditions for mosquito proliferation. The presence of positive sites even during the dry season is a concern as it contributes to malaria transmission year-round.

The majority of the sampled sites were found to contain *Anopheles gambiae* larvae, with only a few containing *Culex* mosquitoes. This indicates that the conditions in these sites were more conducive to the proliferation of *Anopheles gambiae* than other species. *Anopheles* mosquitoes prefer freshwater habitats such as ponds, marshes, slow-moving streams, and irrigated farmland [3]. They thrive in conditions where there is a higher concentration of organic debris, and are more active and capable of breeding at higher temperatures than other mosquito species. In general, the temperature range for optimal mosquito breeding is 20–30 °C [11]. *Anopheles* mosquitoes require high humidity levels to breed and thrive and hence they are more likely to be found in humid regions where there is a lot of rainfall or near stagnant water bodies [8]. These mosquitoes prefer feeding on humans for their blood meal and consequently, they are more likely to be found in areas near human habitation, making them a more significant threat to humans than other mosquito species [26]. *Anopheles* mosquitoes are more active during the rainy season where they often lay their eggs in temporary water sources that form during heavy rainfall, and hence making them more common in regions that experience seasonal precipitation [11]. These conditions were reported in the study area during the period of this study, and therefore could explain the high abundance of *Anopheles* mosquitoes compared to other species.

The co-occurrence of both mosquito genera in the same habitat was also observed in this study. This result is in agreement with previous research that showed that the two genera can coexist in the same habitat despite having different breeding requirements [47, 48]. These findings are consistent with those found in southern Ghana during the rainy season which showed a significant presence of *Anopheles* mosquitoes in urban areas [49]. However, low abundance of *Anopheles* mosquitoes was reported during the rainy season in the Korhogo area of northern Cote d’Ivoire [50], which is in contrast to these findings. It is important to note that heavy rains and floods can wash away the proliferation sites of *Anopheles* mosquitoes, eliminating the mosquito eggs and larvae [51]. In this study, no floods were witnessed in the study areas during the rainy season, which may account for the high proportion of *Anopheles* mosquito larvae observed.

Both *Anopheles* and *Culex* mosquitoes have been reported in Kwale, Kenya with the abundance of *Anopheles* mosquitoes being higher in the rural areas than in the urban areas which is consistent with these findings [52]. This highlights the importance of continuous mosquito surveillance in order to assess the risk of disease transmission and develop targeted mosquito control strategies in this region. The co-occurence of *Anopheles* and *Culex* may have several implications for vector control programmes. *Anopheles* are the main malaria vectors while *Culex* mosquitoes can transmit a variety of diseases such as West Nile virus and filariasis, and therefore the co-occurence of both mosquitoes necessitates for more complex vector control protocols targeted to both *Anopheles* and *Culex* mosquitoes.

This should include a combination of measures such as use of insecticide-treated bed nets, indoor residual spraying, and larval management strategies such as draining stagnant water sources and use of biological mosquito larvicides to eliminate the mosquito larvae. Mosquito surveillance should also be enhanced in this area to determine the abundance of different mosquito species which can help in targeted mosquito control programmes and in monitoring the effectiveness of these measures. Although other malaria vectors have been reported in Kwale before, we could not conclusively ascertain why they were not identified in this study. However, we suggest that their absence could be attributed to factors such as climate change, changes in land use and human population density which are known to influence the distribution of mosquitoes in a given area.

### Physical characteristics of proliferation sites

Most proliferation sites identified were natural in the form of marshes, swamp margins, edges of shallow rivers, roadside pools and animal hoof-prints. This is in agreement with past studies which found that *Anopheles* mosquitoes prefer to breed near human settlements along the edges of shallow rivers, transient roadside puddles, marsh margins, and tree holes [53–55]. Additionally, similar mosquito proliferation sites were discovered in Western Kenya and in Ethiopia [56, 57]. In contrast to these results, Hinne and others [58] categorised the majority of anopheline larval habitats found in Ghana’s three main ecological zones as man-made. The low abundance of artificial mosquito proliferation sites in this region could be explained by the low levels of infrastructural development and less human activities on the environment since the local community is composed of small-scale farmers, pastoralists and traders in a rural set-up.

More sites sampled had mud substrates and were semi-permanent. Faehler and others [59] suggested that the type of soil in a larval habitat and its quality can determine the chances of survival and influence the development of *Anopheles* mosquito larvae. *Anopheles gambiae s.l*. proliferate in habitats with hydromorphic and holomorphic soil substrates due to their ability to retain water for a longer time and also to provide a conducive saline environment for growth of the mosquito larvae [60]. Semi-permanent and temporal mosquito larval habitats were also observed in Western Kenya [61]. This might be because there are fewer predators for the larvae in smaller temporary habitats than in larger permanent habitats [62].

A majority of the sites observed were exposed to full sunlight and had a shallow depth of less than 1 m with an average size of less than 10 m^2^. The growth of algae, a vital source of nourishment for developing mosquito larvae, depends on the presence of sunlight in a larval habitat [62]. Sunlight also warms the water to a suitable temperature that is conducive for growth and development of the mosquito larvae [58, 63, 64]. *Anopheles* mosquitoes prefer breeding in small and shallow water bodies as those observed in this study [51, 53, 56, 57, 65]. Small and shallow water bodies are more suitable for mosquito breeding since they are less vulnerable to water currents and tides which can wash away the mosquito eggs and larvae as compared to large and deep water bodies [66]. These sites are also unsuitable for habitation by other organisms which may be competitors or predators of the mosquito larvae [67]. On the other hand, small and shallow water bodies are more likely to dry faster especially if they are not associated with a larger water body.

The most prevalent type of flora found in the sites was algae. Since algae provides the larvae with nourishment, it was positively correlated with the presence of *Anopheles* mosquito larvae at potential proliferation sites [58, 63]. The proportion of sites with high vegetation cover consisting of algae, emergent and submerged vegetation was very low and none was positive for the mosquito larvae, an indication that the presence of mosquito larvae was inversely correlated to the amount of vegetation in the water body. High levels of vegetation growth generally interfere with light penetration in the water and hence affect the growth of algae and the temperature of the water body [58, 68, 69].

### Physicochemical parameters of the sites

Except for the temperature, which was lower in the positive sites than in the negative sites during the dry season, there was no other noticeable difference between the positive and negative sites during either the dry or rainy seasons. According to this study, there was no apparent difference in temperatures between the dry and rainy seasons. Notably, temperatures reported in both seasons encouraged the presence of *Anopheles* mosquito larvae, and this was consistent with the findings obtained in different places [70, 71]. Although electrical conductivity, total dissolved solids, salinity, and ammonia were significantly lower in the rainy season than in the dry season, there was no evidence that these variables could influence the mosquito larval presence or absence at the sites throughout the two seasons. However, it is notable that the levels recorded for conductivity, total dissolved solids, salinity, and ammonia in both seasons were favourable for mosquito breeding, which was comparable to the findings of a study conducted on a Nigerian university campus [72]. The low levels of these parameters during the rainy season can be attributed to the dilution of environmental surface water by rainwater [65, 71]. However, Emidi and others [47] reported a positive correlation between *Anopheles* mosquito larval abundance, salinity, and conductivity.

Mosquitoes prefer breeding in sites with alkaline pH [11, 73, 74], which concurs with the findings of this study since most of the sites had alkaline pH levels except one negative site, which was slightly acidic. High pH levels in the sites were positively correlated with dissolved oxygen, biological oxygen demand, and nutrients. Dissolved oxygen, biological oxygen demand, pH, and nutrients evaluated in this study were positively correlated and negatively correlated with turbidity. The presence of of nitrates, nitrites, ammonia, and phosphates can be attributed to the use of fertilisers containing ammonium and phosphorus in the farms since most of the sites were adjacent to the farms, while turbidity is associated with silt, mud, algae, and plant pieces [51, 73, 75, 76]. The high level of nutrients has been reported to promote excessive growth of water plants and microorganisms in the water bodies which reduces turbidity of the water making it more suitable for the proliferation of mosquitoes [77, 78].

Similar research in Western Kenya revealed that the amount of nutrients in the proliferation sites had no effect on whether *Anopheles* mosquito larvae were present or absent [79]. Excessive growth and multiplication of microorganisms in water bodies affects their biological oxygen demand and is an indicator of water pollution [76]. Finding *Anopheles gambiae* mosquitoes larvae in polluted water is uncommon since the species is believed to prefer proliferating in clean, unpolluted water in the environment. However, the presence of *Anopheles* mosquito larvae in unclean polluted water has been reported [47], which shows that the mosquitoes could have become more adapted to survive in polluted water to enhance their chances of survival. This might have an impact on how mosquitoes are distributed and abundant in the environment, which would then have an impact on how quickly and frequently malaria spreads.

### Metataxonomic analysis of bacterial communities

In this study, beta diversity analysis revealed that the composition of bacterial communities was not significantly correlated with the presence or absence of mosquito larvae. Instead, the bacterial composition appeared to be influenced by the geographical locations of the sites. These findings align with previous studies that also found no association between bacterial composition and the occurrence or absence of mosquito larvae in potential proliferation sites [5, 76, 78]. However, contrasting results have been reported in other studies, where the structure of bacterial communities in mosquito larval habitats showed a correlation with the presence of mosquito larvae [80, 81]. The differences observed in these studies could not be conclusively explained, but it is hypothesised that the geographical location of the sites may impact the bacterial compositions in mosquito larval habitats. Notably, there was a difference in bacterial communities between sylvatic and domestic proliferation sites of *Aedes aegypti* in Gabon, and these bacterial communities were also found to be correlated with those present in the midgut of adult mosquitoes [82]. This similarity in bacterial community profiles suggests that the origin of bacteria in the sites may be the same for both positive and negative sites.

The findings of this study indicate that the diversity within samples varied significantly between the sites, indicating distinct and independent richness, evenness, and abundance of bacterial communities in each site. Additionally, it was observed that alpha diversities of bacteria were generally higher in the positive sites compared to the negative sites, and these differences were not associated with the evaluated physicochemical parameters. Although the observed differences in alpha diversity between the sites could not be definitively explained, factors such as age, the presence or absence of mosquito larvae, and the physical location of the sites are suggested to influence these variations. These findings are consistent with other studies that have proposed a positive correlation between bacterial abundance and the age of larval habitats [78, 83]. It is also suggested that mosquito larvae can modify bacterial communities in their habitats through feeding or the egestion of bacteria, which could explain the higher alpha diversity in the positive sites [84]. Mosquito larval activities such as feeding and excretion in the habitats may create optimal conditions for the growth of bacteria, which might otherwise go undetected in uncolonized sites where suitable growth environments are lacking [85].

Pseudomonadota, Bacteroidota, and Actinomycetota were found to dominate in all sites, accounting for 96 % of the total reads. These findings align with other studies, where similar bacterial phyla were reported in mosquito larval habitats, such as Firmicutes, Pseudomonadota, and Actinomycetota [80]. These phyla were also observed in the larval habitats of *Anopheles coluzzii* and *Anopheles gambiae* in Cameroon, as well as in three Kenyan Islands in Lake Victoria [79, 86]. Another study conducted in Kenya highlighted the prevalence of these phyla in semi-natural habitats of mosquito proliferation, with Cyanobacteria being the second most abundant phylum [87]. Furthermore, the same phyla were found to be the most abundant in household water-storage containers in India [82]. The higher abundance of Pseudomonadota in mosquito breeding sites suggests that certain species within the phylum may thrive in conditions favourable for mosquito larval development. The exact mechanisms behind this positive association are not fully understood, but it is possible that Pseudomonadota bacteria contribute to nutrient availability, create favourable ecological conditions, or interact with other microorganisms to support mosquito larval growth [88]. Bacteroidota is another phylum that encompasses a wide range of bacteria with diverse functions [89]. In mosquito larval habitats, Bacteroidota bacteria have been commonly detected, and they have been associated with various ecological roles such as nutrient processing. Bacteroidota bacteria are known for their ability to degrade complex organic matter, including polysaccharides and proteins [90]. In mosquito larval habitats, where organic matter accumulates, Bacteroidota bacteria likely contribute to the breakdown of organic materials, releasing nutrients that can be utilized by mosquito larvae. Interactions with other organisms: Bacteroidota bacteria may also interact with other microorganisms present in the larval habitats. These interactions could involve mutualistic relationships, where Bacteroidota bacteria provide essential nutrients or create suitable conditions for other organisms, including mosquito larvae. Actinobacteria, including some members of the phylum Actinomycetota, are well-known producers of bioactive compounds with antimicrobial properties [91]. These bacteria can produce secondary metabolites, such as antibiotics, that can inhibit the growth of other microorganisms [92]. In mosquito larval habitats, actinobacteria may contribute to the natural defence mechanisms against pathogens and compete with other microorganisms for resources. Actinobacteria are also involved in nutrient cycling processes [93]. They play a crucial role in the decomposition of organic matter, releasing essential nutrients that can be utilized by mosquito larvae or other organisms in the habitat.

At the class level, the most common bacterial groups detected were Gammaproteobacteria, Bacteroidia, Alphaproteobacteria, and Actinobacteria, accounting for a total of 76.67 % of all bacteria detected. Gammaproteobacteria was consistently found to be the most prevalent class of bacteria in various studies [94, 95]. Although Bacilli were not among the commonly detected groups in the current study, they were reported as one of the most abundant classes in previous research [94]. Alphaproteobacteria and Cyanobacteria were found to be the most common classes associated with semi-natural mosquito habitats in Kenya [87]. Betaproteobacteria and Alphaproteobacteria were identified as the most abundant bacterial classes in household water-storage containers in India [82], while a study on Kenyan Islands of Lake Victoria found Betaproteobacteria to be the most common class in mosquito larval habitats [79]. Other frequently found classes included Verrucomicrobiae, Planctomycetes, Microgenomatia, Gemmatimonadetes, Acidimicrobiia, Cyanobacteriia, Chloroflexia, and Saccharimonadia.

The most frequently observed bacterial orders were Burkholderiales, Flavobacteriales, Chitinophagales, Sphigomonadales, Micrococcales, Rhizobiales, Sphigobacteriales, Enterobacterales, Frankiales, and Cytophagales. Burkholderiales and Cytophagales have been considered indicator species in water samples collected from the breeding sites of *Anopheles darlingii* [94]. Many of the families detected in this study have previously been associated with *Anopheles* mosquitoes, with the most abundant families being Commamonadaceae, Flavobacteriaceae*,* and Chitinophagaceae [79, 94, 96, 97]. In another study, it was evident that the most abundant families in the larval habitats of *Aedes albopictus* in Italy were Sphingobacteriaceae, Spirosomaceae, Chitinophagaceae, Cellvibrionaceae, Burkholderiaceae, Caulobacteraceae, Planococcaceae, Cytophagaceae, and Blastocatellaceae [98].

The findings from our study provide valuable insights into the association between bacterial populations and positive environmental conditions in relation to mosquito oviposition and habitat selection. The presence of certain bacterial genera, such as *Vibrio* and *Cutibacterium,* with significantly higher abundance at positive sites compared to negative sites suggests their potential role in creating favourable conditions for mosquito oviposition. Conversely, the genus *Methylorubum* exhibited lower abundance in the positive sites compared to the negative site environment. However, studies conducted in Ethiopian mosquito proliferation sites identified *Bacillus, Pseudomonas, Micrococcus,* and *Serratia* as the dominant genera in bacterial genera [99]. Similarly, another study reported the presence of *Rubrivivax, Hydrogenophaga, Rhodobacter, Pseudomonas,* and *Flavobacterium* in mosquito larval habitats in Western Kenya [79]. These bacteria were also discovered in the larval habitats of *Aedes aegypti* associated with domestic water storage containers in Thailand and Laos [69]. The bacterial communities present in mosquito larval habitats may serve as indicator species for high-potential proliferation sites, affecting larval survival, adult fitness, vector abundance, distribution, and ultimately impacting malaria transmission [100]. The findings from Sumba and others [101] provide additional evidence regarding the role of isolated bacteria and their associated volatiles in mosquito oviposition and habitat selection. In this study, various bacterial species, including unclassified *Firmicutes, Aeromonas, Pasteurella, Pseudomonas, Vibrio, Acinetobacter*, and Enterobacteriaceae, were isolated from soil collected beneath oviposition sites and larval habitats. These isolated bacteria were found to restore the attractiveness or stimulant properties of sterile soils, but not filtered distilled water. This result suggests that the presence of microorganisms or volatile organic compounds (VOCs) in water is crucial for mosquitoes to utilize kairomones, which are chemical cues that provide information about their environment [102].

The information obtained about bacterial communities in mosquito larval habitats can be used to design eco-friendly mosquito control methods. For instance, investigating the potential larvicidal effects of specific bacterial communities associated with the sites could help in designing targeted interventions. Disrupting the growth of certain bacterial communities predominant in mosquito larval habitats can render those habitats unsuitable for mosquito breeding. Again, monitoring the bacterial communities in larval habitats can aid in identifying potential breeding habitats and focusing vector control interventions, as the bacterial community can serve as an indicator of the mosquito population present. Considering that bacterial communities in larval habitats may contribute to the vectoral capacity of mosquitoes, these findings provide valuable information for researchers investigating disease transmission mechanisms and developing strategies to curb malaria transmission.

### Limitations of the study

The scope of this study was limited to a specific region within the Kenyan Coast, making it unclear whether the ecological factors associated with mosquito larval habitats can be replicated in different environments. Although other malaria vectors were previously reported in Kwale, this study was unable to conclusively ascertain why they were not identified. Additionally, this study did not examine the specific roles of certain bacteria that were identified as dominant in the larval habitats, nor did it evaluate other factors like interspecific competition and predation in influencing the oviposition response and survival of *Anopheles* mosquitoes.

## Conclusion

In this study, we conducted a comprehensive analysis of bacterial communities in mosquito larval habitats, shedding light on their composition and potential implications for mosquito population dynamics. Our findings revealed the predominance of Pseudomonadota, Bacteroidota, and Actinomycetota across all sites, underscoring their significant roles in these ecological niches. Notably, we identified *Vibrio* and *Cutibacterium* as being significantly more abundant in positive sites compared to negative sites, while *Methylorubum* exhibited lower abundance in the positive sites. These differential abundance patterns suggest a potential association between these bacterial genera and favourable environmental conditions for mosquito proliferation.

Furthermore, our results unveiled a positive correlation between the presence of *Anopheles* mosquito larvae and the rainy season, as well as bacterial abundance. Conversely, we observed negative correlations with several physicochemical parameters, including electrical conductivity, total dissolved solids, salinity, and ammonia. These findings highlight the intricate interplay between environmental factors, bacterial communities, and mosquito oviposition and population growth. Importantly, our study highlighted the influence of temporal and geographical factors on the structure of bacterial communities in mosquito larval habitats. By comparing our findings with previous studies, we gained valuable insights into the dynamic nature of bacterial compositions and their associations with mosquito populations. This knowledge holds promise for predicting potential proliferation sites based on the physicochemical properties and bacterial community compositions of environmental water samples.

Overall, our study advances the understanding of bacterial communities in mosquito habitats, providing crucial information for the development of targeted mosquito control strategies and the elucidation of mechanisms underlying mosquito-borne disease transmission. Moving forward, investigations focusing on the temporal dynamics of bacterial communities and their impact on mosquito populations will further enhance our ability to predict and mitigate mosquito-borne diseases, ultimately contributing to improved public health outcomes.
